# Advanced IoT Pressure Monitoring System for Real-Time Landfill Gas Management

**DOI:** 10.3390/s23177574

**Published:** 2023-08-31

**Authors:** Cormac D. Fay, John P. Healy, Dermot Diamond

**Affiliations:** 1 SMART Infrastructure Facility, Engineering and Information Sciences, University of Wollongong, Wollongong, NSW 2522, Australia; 2 CLARITY: Centre for Sensor Web Technologies, Dublin City University, Glasnevin, D09 V209 Dublin, Ireland; 3 National Centre for Sensor Research (NCSR), Dublin City University, Glasnevin, D09 V209 Dublin, Ireland; 4 Insight Centre for Data Analytics, Dublin City University, Glasnevin, D09 V209 Dublin, Ireland

**Keywords:** landfill gas, gas pressure measurement, environmental monitoring, Internet of Things (IoT), environmental management

## Abstract

This research presents a novel stand-alone device for the autonomous measurement of gas pressure levels on an active landfill site, which enables the real-time monitoring of gas dynamics and supports the early detection of critical events. The developed device employs advanced sensing technologies and wireless communication capabilities, enabling remote data transmission and access via the Internet. Through extensive field experiments, we demonstrate the high sampling rate of the device and its ability to detect significant events related to gas generation dynamics in landfills, such as flare shutdowns or blockages that could lead to hazardous conditions. The validation of the device’s performance against a high-end analytical system provides further evidence of its reliability and accuracy. The developed technology herein offers a cost-effective and scalable solution for environmental landfill gas monitoring and management. We expect that this research will contribute to the advancement of environmental monitoring technologies and facilitate better decision-making processes for sustainable waste management.

## 1. Introduction

One contributing factor to the pressing issue of the climate crisis is the management of waste, which accounts for approximately 5% of the global greenhouse gas contribution [1,2]. According to the World Bank, the global waste generation is projected to increase by approximately 30% (2.59 billion tonnes) by 2030 and 70% (3.4 billion tonnes) by 2050 [3]. Effective waste management is crucial, and landfilling remains a popular and economical method globally—accounting for 25.2% of global treatment and waste disposal [4,5]. However, landfilling can contribute to greenhouse gas emissions and other environmental concerns [6,7,8,9].

Monitoring landfill sites is essential due to the environmental impacts they can have. Issues such as leaching of hazardous substances, underground fires, and greenhouse gas emissions have been well-documented [10,11,12,13,14,15]. In densely populated areas, landfill sites pose a public health risk and limit city expansions [16,17,18]. Modern landfill site designs focus on controlling and treating emissions from the waste body, highlighting the need for effective monitoring systems [19,20].

Gas pressure sensing devices offer the potential to improve the detection and management of landfill gas emissions. Landfill sites are equipped with gas extraction networks connected to a main flare. Supervisory control and data acquisition (SCADA) systems analyse and treat the gas produced. However, the transient nature of landfill gas production and the limitations of manual sampling programs make actual physical measurements challenging [21]. Autonomous monitoring systems that can measure gas pressure levels and provide reliable data to landfill managers are crucial for effective gas management and early detection of events such as flare shut-down/failure or potential blockages and pressure increases [22].

The effective monitoring and management of landfill sites are necessary to minimise risks to public health and the environment. Studies have demonstrated the accumulation of landfill gas and migration events due to maintenance issues [23]. Incidents resulting from poor waste management practices have caused property damage, loss of life, and environmental and health hazards [24,25,26,27,28,29,30,31]. To mitigate these risks, monitoring practices must be in place.

The past two decades have seen progress in the development of autonomous systems for landfill gas monitoring. Several works have investigated the deployment of IoT gas monitoring solutions on active landfill sites [32,33,34]. However, the focus has mainly been on monitoring the major greenhouse gas constituents, CO${}_{2}$ and CH${}_{4}$. The use of costly sensors for these targets can impact scalability and the ability to detect localised disturbances such as blockages along the extraction network.

Gas pressure along the extraction network is another critical factor contributing to hazardous events. Excessive weight of the waste body or high moisture content within the gas can impede or stop gas extraction, resulting in high localised pressures that are difficult to detect with conventional manual measurements [22]. Therefore, spatially distributed pressure sensors offer a more effective and cost-effective means of improving site management and preventing such events.

Previous work has demonstrated the feasibility of wirelessly monitoring the major chemical constituents of landfill gas (CO${}_{2}$ and CH${}_{4}$) using an autonomous system [35,36,37]. Building on this foundation, we present the development of a remotely deployable pressure sensing platform for the continuous monitoring of gas extraction network pressure, with data wirelessly transmitted to a central base station and displayed in real time. In this study, we report on a field deployment lasting approximately three months in three deployment locations to evaluate the performance of our prototype system. Through analysis of the collected data, we demonstrate the system’s capability to detect events such as network shut-downs in real time and compare the results to those from the landfill’s SCADA system. Our system offers a significant improvement over conventional manual measurements and can help prevent hazardous events resulting from pressure build-up in the gas extraction network.

## 2. Background

To provide a comprehensive understanding of the context and existing knowledge related to landfill gas monitoring, we present relevant background literature in this section. The literature highlights the environmental and health risks associated with poorly managed landfill sites and emphasises the importance of effective monitoring and management practices. Studies investigating the deployment of gas monitoring solutions on landfill sites are discussed, with a focus on the monitoring of major greenhouse gas constituents. We also address the limitations and challenges associated with manual sampling programs and the need for autonomous monitoring systems. Finally, we highlight the significance of gas pressure along the extraction network as a critical factor contributing to hazardous events.

Franzidis et al. [23] conducted a study at a landfill site in Montreal, Canada, over a three-year period. The research demonstrated that the accumulation of landfill gas was influenced by seasonal changes, surface conditions, and atmospheric conditions. The study also highlighted the occurrence of significant migration events due to the maintenance of the site’s gas collection system. These findings underscore the importance of effective monitoring and management practices to minimise risks to public health and the environment.

Various incidents resulting from poor waste management practices have demonstrated the potential consequences. For instance, Drouin et al. [24] reported significant property damage and loss of life due to the accumulation and explosive effects of CH${}_{4}$ gas. The Loscoe methane gas explosion in the UK and the Skellingsted Landfill incident in Denmark are examples wherein atmospheric pressure drops, heavy rain, and low permeability of topsoil led to hazardous outcomes [25,26]. Additionally, environmental and health hazards resulting from landfill incidents have been well-documented [27,28,29,30]. These incidents highlight the need for effective monitoring and management practices to mitigate risks associated with landfill sites.

The existing literature has investigated the deployment of gas monitoring solutions on landfill sites, with a focus on major greenhouse gas constituents. Studies by Mabrouki et al. [32], Venkatesan et al. [33] and Reddy et al. [34] explore the use of IoT-based gas monitoring systems for tracking CO${}_{2}$ and CH${}_{4}$ emissions. However, the costliness of the sensors for these targets can limit scalability and the ability to detect localised disturbances along the extraction network.

Manual sampling programs, commonly used for landfill gas monitoring, have limitations in terms of cost, personnel, time, and access [21]. Autonomous monitoring systems that can measure gas pressure levels offer a more effective and cost-effective alternative. Gas pressure along the extraction network can indicate potential blockages, pressure increases, and other events that conventional manual measurements may not detect [22].

There have been several notable contributions that have focused on different aspects of monitoring and mitigation. For example, Aziz and Zahra [38] presented a prototype design of a landfill gas pipe leak monitoring system using the Internet of Things (IoT) approach. Their work primarily focused on detecting gas leaks in the pipeline using gas sensors and transmitting the data to a central base station. While their system addresses the important issue of gas leaks, our research expands on this by developing an advanced IoT pressure monitoring system for real-time gas management [39]. We not only monitor gas leaks but also provide continuous measurement of gas pressure levels, enabling the early detection of critical events and facilitating more effective gas extraction network management.

Another relevant study by Saha et al. [40] proposed waste management using the Internet of Things. Their work emphasised waste reduction, recycling, and composting techniques to minimise waste generation and promote sustainable practices. In comparison, our research focuses specifically on landfill gas monitoring and management. We have developed a stand-alone device capable of autonomously measuring gas pressure levels on active landfill sites, enabling real-time monitoring of gas dynamics and early detection of critical events [34]. Our device incorporates advanced sensing technologies, wireless communication capabilities, and data transmission systems for remote access and improved landfill management.

In summary, the background literature underscores the environmental and health risks associated with landfill sites and emphasises the importance of effective monitoring and management practices. Existing studies have focused on monitoring major greenhouse gas constituents, CO${}_{2}$ and CH${}_{4}$, but there is a need to address the limitations of manual sampling programs and explore alternative monitoring approaches. Gas pressure along the extraction network emerges as a critical factor contributing to hazardous events, highlighting the importance of autonomous monitoring systems.

## 3. Experimental

### 3.1. Sensing Strategy

**Figure 1:**
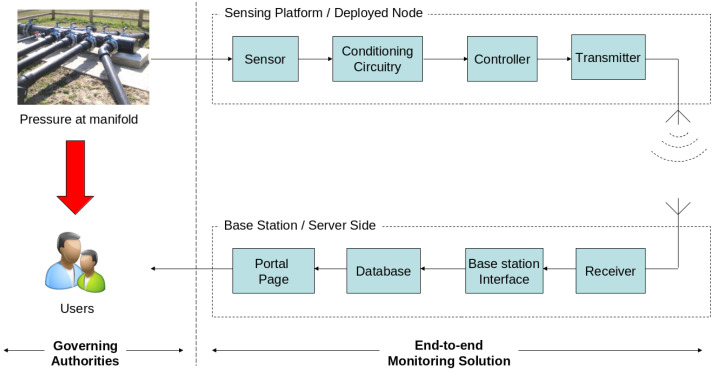
Visual representation of the autonomous pressure sensing system architecture. The model shows the progression of sensed pressure data from the manifolds to the users via an on-line portal page. The model also shows an alternate data route (red arrow) which represents an encapsulated data transfer approach, i.e., hiding the more complex low-level workings of the system.

Figure 1 shows the high-level approach adopted to harvest pressure information from the landfill manifold, through to retrieval of on-line data via a web-accessible portal page. The pressure sensor with appropriate signal conditioning circuitry is linked into a communications infrastructure to deliver data remotely to a database, from which the data can be displayed in a graphical format by relevant stakeholders.

### 3.2. Location Dependencies

The location of landfill sites is typically chosen to be at safe distances away from populated areas to minimise potential risks and ensure compliance with environmental regulations. As a result, these sites may not be high-priority areas for the deployment of advanced communication infrastructure such as 3G, 4G, or 5G networks. Consequently, the signal strength and coverage provided by service providers may be limited in these locations.

During our research, we explored access to multiple landfill sites for our experimental deployments. However, only one landfill site agreed to participate in our study. Interestingly, this particular site was situated in an area where no communication signals other than GSM (calls/text) were available. This presented a unique challenge as our monitoring system required reliable communication capabilities for data transmission.

To address this challenge, we had to consider additional energy requirements to ensure that our system could operate autonomously for extended periods without the need for frequent maintenance or battery changes. This location dependency played a crucial role in our system design, as we needed to optimise energy consumption and ensure the longevity of the monitoring device in areas with limited communication infrastructure.

By factoring in these location dependencies and considering the specific challenges posed by the chosen landfill site, we were able to develop a robust monitoring system that could effectively operate in remote areas with limited communication resources. These considerations highlight the importance of tailoring the system design to the unique conditions and requirements of each landfill site, thereby enhancing the reliability and effectiveness of the monitoring solution.

### 3.3. Calibration

To ensure that the sensor was producing a reliable signal, it was tested using a clean power supply (TTi EL302Tv; Thurlby Thandar Instruments, Cambridgeshire, UK) with a 10 V source in accordance with the product data sheet. The potential difference between the two signal pins was measured using a bench top multimeter (TTi 1604; Thurlby Thandar Instruments, Cambridgeshire, UK) whereupon a vacuum was applied to the sensor through securely fixed tubing. The intensity of the vacuum applied was varied between 0 and −100 mBar by adjusting the vacuum source. In addition to this, the vacuum source was replaced with a positive pressure source and, in the same manner, the positive applied pressure was varied between 0 and +100 mBar. For both processes, the voltage difference was recorded by the multimeter and the readings were saved to file on a PC via an RS232 interface using the instrumentation software. In addition, a verified instrument (Digitron PM-10; Margam, Port Talbot, UK) was used in parallel to investigate the sensor response to a reference system.

### 3.4. Signal Conditioning

**Figure 2:**
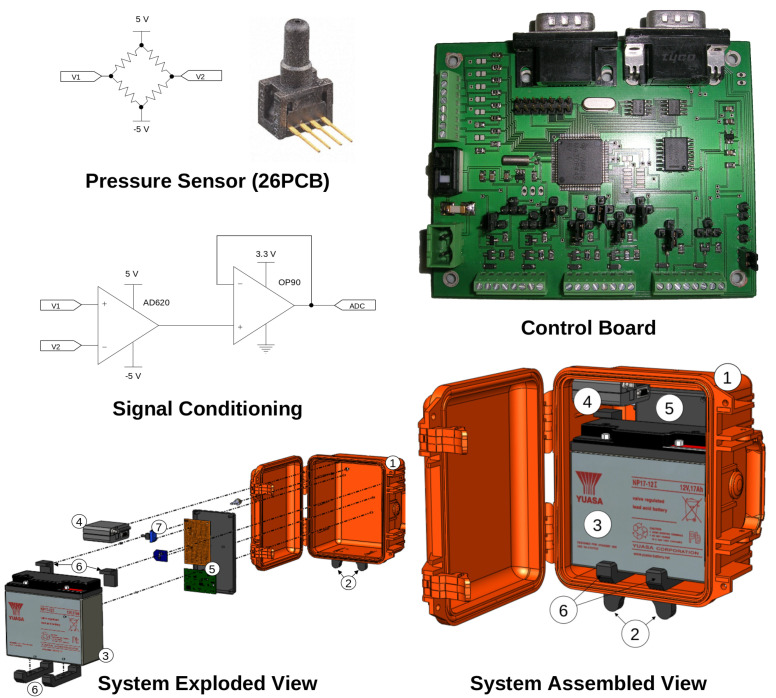
System design. **Top Left**: Schematic of the analogue signal conditioning circuitry implementation. **Top Right**: Captured image of the control board. **Bottom**: CAD models of the sensing platform. (1) Peli Case. (2) External Mounting Bracket. (3) Battery. (4) GSM Module. (5) Control and Signal Conditioning Boards. (6) Battery Mounting Brackets. (7) Pressure Sensor.

Figure 2 (Top Left) presents the schematic of the analogue conditioning circuitry designed to translate the output signal generated by the sensor into a suitable format for measurement via the microcontroller ADC channel (12-bit, MSP430F449; Texas Instruments, Dallas, TX, USA). A 5 V power supply was enabled via a linear voltage regulator (LM1085; Texas Instruments, Dallas, TX, USA) in addition to a DC/DC voltage inverter (ICL 7600; Intersil Corporation, Milpitas, CA, USA) to supply −5 V. The potential difference allowed for 10 V to power the pressure sensor (Honeywell 26PCBFA1G; Charlotte, NC, USA). The Honeywell 26PCBFA1G sensor is a miniature low-pressure sensor designed for use with wet or dry media, and was placed within the platform, as seen in Figure 2. It incorporates a specialised piezoresistive micromachined sensing element, featuring four active piezoresistors forming a Wheatstone bridge, as seen at the top left of the figure. This sensing technology offers high performance, reliability, and accuracy. The sensor provides a millivolt output signal that is proportional to the input pressure. With a pressure range capability from 1 psi to 250 psi, the 26PCBFA1G sensor accommodates a wide variety of wet and dry media. It operates within a temperature range of −40 °C to 85 °C. The sensor’s construction is in compliance with RoHS standards and is designed and manufactured according to ISO 9001 standards [41].

The two signal pins (V${}_{1}$ and V${}_{2}$) of the sensor were connected to the inverting and non-inverting inputs of an instrumentation amplifier (AD620), respectively, and the signal gain was controlled using a variable resistor (trimmer). This resulted in a signal output of ca. ±1.1 V corresponding to a stimulus of ±100 mBar. A second trimmer to the instrumentation amplifier allowed for signal offset in order to ensure that the full signal was positive in order to ensure compatibility with the microcontroller ADC measurement range (0–3.3V). This resulted in a signal range from ca. 0.4V (−100 mBar) to 2.6 V (+100 mBar). To optimise conditioning, the signal was passed through a voltage follower (OP90) to prevent the signal from exceeding the microcontroller ADC measurement range. Overall, this output signal covered the target sensing range and allowed for a comfortable buffer with an overall sensing range from −163 mBar to +136 mBar. The calibration routine described previously (Section 3.3) was repeated twice: (a) to investigate the response range of the conditioning circuitry verifying signal linearity, and (b) to ensure that the ADC channel was viable and additionally develop a conversion model to mBar.

### 3.5. Measurement Procedure and Data Handling

The selection of an appropriate sampling rate was a crucial aspect of our measurement procedure. It directly influences the accuracy of data collection, power consumption, and the duration of deployment without the need for maintenance. In consultation with the landfill site manager, we determined a sampling rate of 4 min for our gas monitoring system. This decision was based on several factors. Firstly, we had the opportunity to place our monitoring system adjacent to the landfill’s SCADA system, which sampled data every 10 min. To ensure compatibility and enable meaningful comparisons, we aimed to select a sampling rate that aligned with the existing practices at the landfill. Additionally, we considered the Nyquist–Shannon sampling theorem, which states that a minimum of twice the sampling frequency is required to accurately represent the signal. This satisfied this theorem while balancing the trade-off between capturing critical events and minimising power consumption.

Figure 2 (Bottom) shows the platform CAD design, which included a GSM module (Siemens MC35iT) for wireless communications via SMS. It was agreed with the landfill management that an hourly reporting frequency was to be established as a minimum for this initial study. Moreover, to maximise the text message space, the microcontroller was programmed to repeatedly wake from sleep mode every 4 min, take a pressure measurement, and transmit data at hourly intervals. The text message was received at an identical GSM module connected to an SMS server. Here, a custom written program was employed to accept newly arrived texts, parse the information, and upload the data onto a MySQL database. The data were further backed up on an additional database server to ensure data backup/redundancy. Access to this server was fast and secure, and users were capable of visualising the data via the on-line portal page.

### 3.6. Deployments

**Figure 3:**
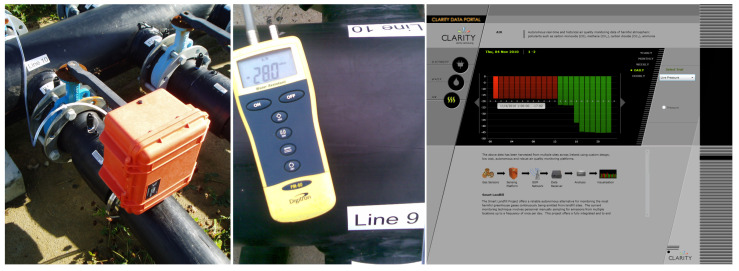
Captured photos of the deployed sensing system (**left**) and a pressure spot check with a reference instrument (**middle**). Screen grab of the sensor portal web application showing a portion of the captured pressure data (**right**).

A 4 week deployment period was initially agreed with the project partners in order to pilot this system and fulfil the requirements of the project. This was successfully deployed and designated as Location #1. Figure 3 (left, middle) presents captured images of the system on deployment in addition to a spot check. After this, the project partners requested a second more active area for monitoring for an additional 4 weeks, which is designated as Location #2. Later, an opportunity arose whereby the main flare was to be turned off to carry out a full service from 12:00 to 16:30. The Environmental Protection Agency (EPA) requested the system be deployed for a third trial at Location #3 in order to examine whether it could detect the shut down and act as an early warning system in the future.

### 3.7. Data Presentation

The portal page, as seen in Figure 3 (right), was written using Microsoft’s Silverlight programming package. The data were presented graphically by a bar chart for hourly reading. Furthermore, the bars are designed to change colour, i.e., green if the pressure is less than −20 mBar (this value can be adjusted to suit the target sensing area or user) and red if greater to indicate a problem. The system has scope to remotely alert site personnel or any other authority if potential problems or issues are detected. Finally, the user may select a yearly, monthly, weekly, daily, or hourly view of the data where desired. Another feature available to users is that, when a single bar is selected, more specific details of that data packet becomes available, e.g., date, time, and pressure levels during that time period.

## 4. Results and Discussion

### 4.1. System Calibration

**Figure 4:**
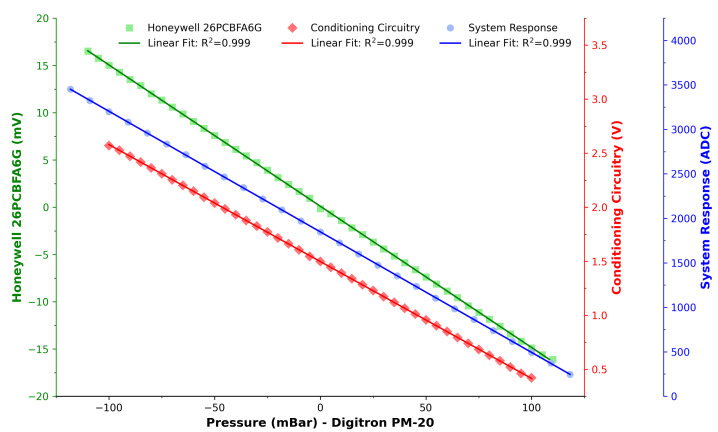
Calibration plot of the sensor (green), conditioning circuitry (red), and system response (blue). Excellent linear fits were achieved (R${}^{2}$ = 0.999) for all responses.

Figure 4 presents the results of the calibration routine described previously in Section 3.3 and Section 3.4. Excellent linear fits (R${}^{2}$ = 0.999) were achieved for all three calibration routines. The direct measurement of the pressure sensor (green, squares) ensured the validity and linearity of the sensor, which was a necessary precursor. The conditioning circuitry plot (red, diamonds) ensured that the designed analogue conversions were sound and its range rests in the positive domain. Finally, the third calibration (blue, circles) involved measurements by the micro-controller’s ADC channel and yielded further reassurance, linearity, and validity of the full system. This also enabled the establishment of a conversion formula from ADC to pressure in mBar as per Equation (1).

(1) $$\mathrm{Pressure}\;\left(\mathrm{mBar}\right)=\frac{1848.3-ADC}{13.6}$$

### 4.2. Deployment Data: Location #1

**Figure 5:**
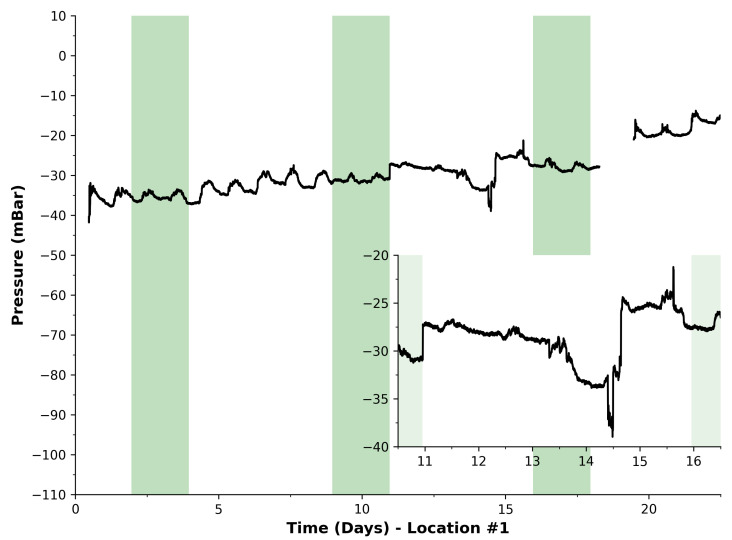
Pressure data harvested during deployment at Location #1. Green regions represent the weekends. Black line is pressure measurements from our device.

Figure 5 presents the data collected during the first two deployment trials for approximately 1 month each. Location #1 data are shown in Figure 5. This was the first deployment of this system, demonstrating the capability of the device to measure pressure data on a landfill extraction network remotely. It can be seen that there was a slight linear drift over the first half of the deployment (i.e., before day 11), from −35 mBar to −30 mBar, superimposed with a repetitive pattern that may to correspond to daily cycles, which is supported by five undulations during the weekdays, e.g., week 2. While the weekends are not as pronounced, this could also be due to daily temperature effects. This pattern ends on day 11, where nearby landfill activity began, which is magnified in the inset. During this time, dumping of new waste material in a nearby cell caused an impact in these measurements. Specifically, the change occurred on Monday morning, which is apparent at the start of week 3, see inset. It was interesting that this detection was possible as the active cell was a secondary connection to the extraction line. This shows promise as certain activities can propagate across the network and be detectable even if it was not a primary connection. It must be noted that the cellular service account was depleted on day 18 and resulted in a loss of reported data to the server. This took place just after weekend 3 and was rapidly corrected on Monday. Fortunately, this allowed the team to ensure that the cellular account was sufficiently credited for subsequent deployments.

### 4.3. Deployment Data: Location #2

**Figure 6:**
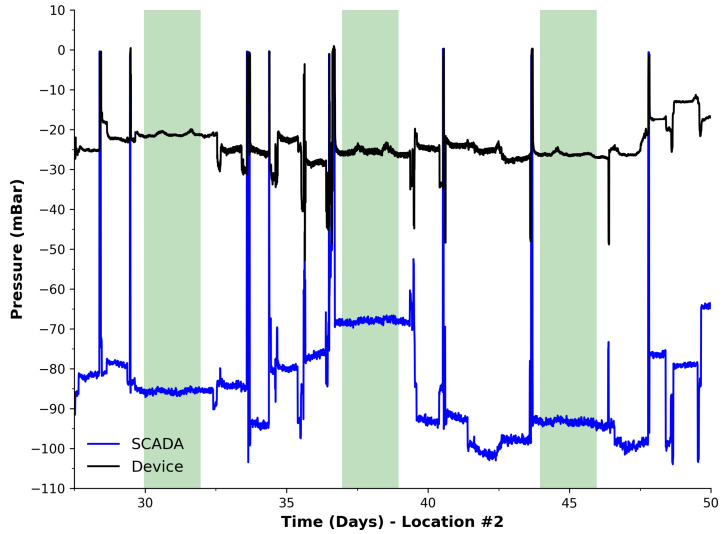
Pressure data harvested during deployment at Location #2. Green regions represent the weekends. Black line is pressure measurements from our device. Blue line is pressure measurements from the site’s SCADA system at the flare.

Given that the system demonstrated a capability of detecting an event at neighbouring cells, it was deployed for a further month at a second location—this time in close proximity to an active cell (Location #2). It must be noted that the system underwent a spot check before deployment, which is pictorially shown in Figure 3 (middle) as −28 mBar. Comparably, our device reported a pressure level of −28.02 mBar, which suggests a high degree of accuracy of the system’s measurements. The captured data during this period are presented in Figure 6. During the Location #2 deployment phase, the SCADA system data at the flare were available and given to us by the landfill project partners, which appears as the blue line in the plot. It can be seen that this dataset shows more activity via a number of noticeable events, resulting in more rapid and pronounced changes in pressure. Overall, a number of events are recognisable in both the device and SCADA data similar to Location #1, but more pronounced and visually evident. It is also interesting that these events exclusively take place during weekdays, with no events appearing during weekends. This supports conceivable observations that events/disruptions to the pressure network are being caused by site activity. A method to detect these events will be explored in the data-processing section (see Section 4.5), which could form the basis of automatic identification through known signal signatures, e.g., flare failure or local blockages along the extraction network, using the site’s SCADA data as reference. Although the SCADA data measurements are at the flare only, they do allow for the event times to be correlated with site activities.

For this study, an event is defined as the time with which the pressure changes suddenly and significantly and later returns to the previous baseline. Specifically, a pressure change of 10 mBar was established to represent a significant event, which was based on the recommendations, experience, and expertise of the site manager and team. A significant change in differential pressure relates to ±10 mBar/4 min (4 min is the sampling period) or greater in magnitude in this case; this was chosen to sufficiently represent an event for this sensing location. In addition to this, descriptors were compiled for each classified event to include the polarity (initial increase/decrease) of the relative change in pressure, start time, end time, duration, magnitude, and all the data points that make up the event. Table 1 presents a summary of detected events for the deployment at Location #2 for both our sensing system and the SCADA system. It can be seen that our device was capable of detecting more events than the SCADA system, which could be due to the higher sampling rate wherein brief events can be missed. This is supported by the detected minimum duration of events matching the sampling periods. Both systems are in good agreement on the maximum duration, notwithstanding the sampling period difference. The average event duration is also in good agreement, which yet again can be impacted via the higher resolution of our system.

### 4.4. Deployment Location #3

**Figure 7:**
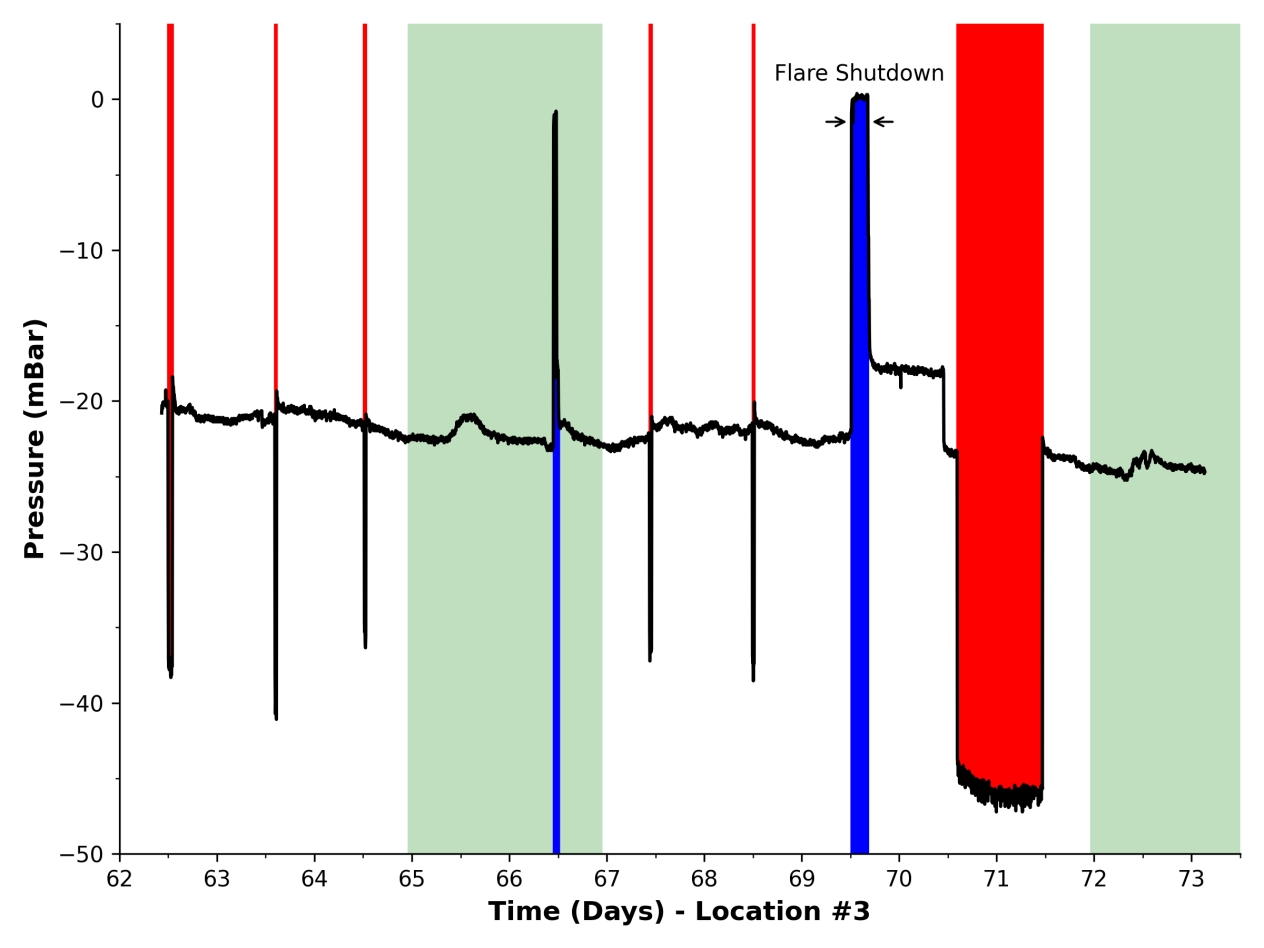
Pressure data harvested at Location #3. Green regions represent the weekends. Black line is pressure measurements from our device. Analysis detected 8 events in total. Blue: events with loss of vacuum pressure. Red: increase in vacuum pressure. The flare shut-down event was detected and indicated as the seventh event in this deployment period.

During phase #2, the main flare was scheduled to undergo necessary maintenance. This was an opportunity to observe whether the developed event analysis program was capable of detecting the shutdown and therefore act as an early warning system for automatic identification. Figure 7 presents the harvested data for this deployment period. Using the developed analysis, eight events were detected and indicated via filling under the blue curve for a reduction in vacuum pressure and red for an increase. The time period when the flare was manually shut-down is identified clearly in the figure as the second blue region or seventh event, i.e., during week 2 on day 69. This event greatly exceeds any previously encountered events which potentially enables flare shut-down/failure to be distinguished from other events encountered. By considering both the magnitude of the pressure (ca. 0 mBar) and the duration of the event, an automatic processing algorithm could be easily developed to detect such a flare shut-down/failure and possibly trigger an alert. The eighth event during day 70 is indicated by the red region (increased pressure), which indicates the time when the flare was primed after the shut-down to ensure that it was operational and to compensate for any untreated gas generated during the shut-down. This event is considerably longer in duration than the shut-down (21 h) and opposite in direction/polarity relative to the normal baseline. Therefore, it appears that this type of event can also be easily identified and distinguished from flare shut-down.

### 4.5. Data Analysis

#### 4.5.1. Global Summary Statistics

Initially, the entire dataset was considered and processed to produce global information, summarising the total number of measurements and the maximum/minimum/average-sensed values. In addition, the number of measurements above an established threshold (−20 mBar—recommended by site management) was also compiled and expressed as a percentage of the total number of samples. Table 2 presents our summary statistics for the entire dataset. The density of measurements (i.e., 20,043 over ca. 2 months) already show that using manual sampling is not feasible for this type of monitoring, whereas our approach is inherently scalable. Furthermore, 67% of detected “events” were less than 1 h in duration, which implies that localised events are much more difficult to detect by the current standard of manual sampling, and therefore this speaks to the need for this technology. The data also show that pressure changes rapidly, over a wide range, with a minimum reading of ca. −53 mBar and a minimum of ca. 1 mBar with an average value of ca. −26 mBar of the entire duration. Finally, it appears that for 13.6% of the total recorded measurements, the pressure levels were above the recommended threshold (−20 mBar) for this extraction line.

#### 4.5.2. Event Analysis

From these descriptors, a summary information set was compiled and is presented in Table 3. It can be seen that 21 events in total were detected during our entire deployment period and that the event durations ranged from as short as 4 min (due to the set sampling period) to 21 h. The average time of these detected events was estimated as ca. 2 h. The causes of these events are unclear and are difficult to determine without metadata relating the events to site activities and factors external to the site (e.g., weather patterns). However, there are two events that can be related to specific site activities, i.e., the flare shut-down and the priming of the system afterward. These two events can be distinguished through their established polarity descriptions. Visual analysis of the previous figures shows that, for this deployment, the longest duration corresponds to priming of the system after the flare shut down, i.e., 21 h (Table 3). The detection of the event of the longest duration, i.e., by ranking the event dataset by its duration, identified an event of decreasing pressure polarity occurring from day 70 to 71. This event (based on polarity, time of occurrence, and duration) corresponded to the priming of the system after the flare shut-down (see Figure 7). Similarly through the same criteria, the maximum event duration with an increasing pressure polarity was identified as taking place on day 69, which corresponds with the duration when the flare was shut-down for maintenance (see Section 4.4). Therefore, it would seem that even simple analytical approaches, as employed in this study, can enable important events to be detected and classified.

#### 4.5.3. Sampling Frequency

Firstly, it is important to note that legislation governing landfill monitoring, such as the United States EPA’s New Source Performance Standards (NSPS) for Municipal Solid Waste (MSW) Landfills [42], the European Union directives [43], and others [44], typically require quarterly monitoring during the active state of the landfill and for an extended period after closure. However, it is worth mentioning that monitoring frequencies may vary based on factors such as landfill size, waste types accepted, and potential environmental impact, and can sometimes include monthly measurements.

Furthermore, it is crucial to consider the distinction between global and local monitoring. While some existing systems, such as the SCADA system, may provide global monitoring at select points, they may miss important events occurring in localised areas. Our portable system, on the other hand, offers the flexibility to be deployed at various locations along the landfill network, providing real-time monitoring and automated alerts for unforeseen events. This local monitoring capability is especially valuable in detecting gas leaks, fugitive emissions and identifying blockages in specific sections of the gas extraction network.

During our project, the opportunity to deploy the system at the flare allowed us to monitor the landfill site on a global basis, albeit at one point along the network. During Deployment Location #2, we found that the SCADA system missed a number of events in the short monitoring period of 3 weeks, allowing one to suspect if it has missed others during the years. This, in turn, brings into question whether 4 min is sufficient. A higher sampling rate, such as 1 min or 30 s, would result in a significantly larger amount of data collected over time. This can pose challenges in terms of data storage, transmission, cost, and processing. It may require more resources and infrastructure to handle and analyse the increased data volume, such as:

- Power consumption: A higher sampling rate would lead to increased power consumption by the monitoring system. This could reduce the overall deployment period without the need for maintenance, as more frequent sampling would require more frequent battery replacements or recharging.
- Data processing and analysis: A higher sampling rate would generate a larger number of data points, potentially requiring more computational resources for data processing and analysis. This could impact the efficiency and speed of data analysis, as well as the complexity of algorithms used for event detection or anomaly identification.
- Specific event detection: A higher sampling rate may enable the detection of more specific events or transient phenomena that occur within shorter time intervals. This could provide additional insights into the dynamics of landfill gas emissions and enhance the understanding of critical processes. However, it is essential to carefully assess the trade-off between the benefits of higher temporal resolution and the associated challenges mentioned earlier.

In comparison to the regulatory standard of four measurements per year, our system’s sampling rate of 4 min provides a significant increase in event detection capabilities. The higher temporal resolution allows for the identification of critical events, such as blockages in the gas extraction network and localised pressure build-up, which can lead to hazardous conditions like underground fires.

Ultimately, the choice of sampling rate depends on the specific objectives of the monitoring system, the desired level of detail in capturing events, the available resources, and the practical constraints of deployment duration and power consumption.

### 4.6. Reported Device Comparison

Our work acknowledges the importance of not only enhancing the sampling rate but also the need to improve upon existing methodologies. While previous works by Mabrouki et al. [32], Venkatesan et al. [33], and Fay et al. [35] have included additional sensors and features, our emphasis on real-time pressure monitoring fills a significant gap in the existing landscape. Pressure, as a physical parameter, plays a pivotal role in forming hazardous conditions, such as underground fires [45]. Our focus on pressure monitoring, which is often overlooked, enables the early detection of critical events, such as flare shutdowns, blockages, and pressure changes, thereby contributing to the safety and efficiency of landfill operations.

To facilitate a clear comparison between our work and the previous research, we have provided Table 4, which outlines key criteria including the sensing environment, sensory targets, monitored pressure, calibration/validation, data comparison to in-field instrumentation, wireless communication methods, electronics platforms, and sampling periods. The table underscores the distinctive aspects of our contribution, particularly our focus on pressure monitoring as a critical parameter for landfill gas management.

While chemical sensors are indeed crucial for monitoring gas constituents, they often exhibit drift over time and require regular calibration. In contrast, pressure sensors offer reliability, stability, and lower power consumption, extending the operational lifespan and sampling frequency of our device. Furthermore, the risk associated with certain chemical sensors in combustible environments, such as those with heating elements, can be mitigated by the use of pressure sensors, which employ simple resistance measurements for determination.

In the context of localised events, such as blockages in the extraction network, pressure sensors become invaluable. These events can go unnoticed by global SCADA analysis systems and lead to hazardous conditions like underground fires. Pressure sensors can detect negative pressure resulting from blockages, thus providing an early indicator of potential issues. Unlike chemical sensors, which can read zero in the presence of a blockage, pressure sensors will exhibit noticeable changes, highlighting the need for attention.

A notable advantage of pressure sensors is their cost-effectiveness and suitability for scaling. Implementing a network of sensors to monitor gases like CO${}_{2}$ and CH${}_{4}$ across a landfill network can be costly and may not effectively address the specific issue of localised events. Our cost-effective pressure sensor solution, explored through this pilot study, offers a targeted approach to address this critical concern in waste management systems.

### 4.7. System Scope

#### 4.7.1. Additional Sensory Targets

It is clear that waste management is a complex issue and the generated gas from the breakdown of said waste can be composed of various chemical species. As a result, it is desirable to autonomously monitor key gas constituents and issue alerts when negative events take place for preventative action to be enacted. Moreover, the more sensory information available, the more likely it is to enable predictions. It is therefore desirable to equip systems with additional sensory targets to suit the target environment. For Example, Mabrouki [32], Venkatesan [33], and Fay [35] enabled the detection of CO${}_{2}$ and CH${}_{4}$, amongst others individually listed in Table 4.

As a consequence of this real-world need, we incorporated scope into our platform design. Figure 2 (top right) presented a captured image of our ad hoc control board. Here, one can see a number of readily available terminals at the user’s disposal. For instance, the bottom wire terminals allow for the choice of standard power supply voltage levels, i.e., 12 V (battery voltage), or a regulated 5 V or 3.3 V. These offer power control switching (Onsemi, FDV304P) for low power consumption. Overall, this board allows for the possibility to power up to 12 devices. The top-left terminals allow for up to eight sensory inputs (12-bit ADCs or I/Os) with in-built potential dividers where desirable to accommodate numerous commercially available sensors. For instance, many sensory modules are readily available that require power as input and outputs a signal line ready for measurement and implementation by our control board. Further customisation is possible through powering signal conditioning circuitry for more specialised sensory requirements.

Specifically upon examination of the sensor modules reported by Mabrouki et al. [32], i.e., MiCS-6814 (NO${}_{2}$ and CO), MQ-7 (CO), MQ-4 (CH${}_{4}$), MQ-136 (H${}_{2}$S), and ME2-O2 (O${}_{2}$), we can verify that our control board is capable of immediately accommodating such sensors. Other targets may also be of interest such as the presence of sulfer compounds [46,47], which can contribute to the generation of highly hazardous pollutants. Existing sensor modules such as the Gravity SO${}_{2}$ Sensor (DFRobot, SEN0470) can readily interface to our system and offer further advantages for enhancing the sensing system for enhancing waste management. Overall, this gives our platform excellent scope for monitoring a number of additional sensors for monitoring desirable gas constituents—in addition to pressure explored in this study.

#### 4.7.2. Other Waste Disposal Methods

While landfilling has been a widely adopted waste disposal technique, particularly in developing countries [48], it is important to recognise the diverse landscape of waste management methods. In addition to landfilling, methods such as incineration/combustion, recovery and utilisation, plasma chemical conversion, composting, energy recovery, and waste reduction strategies have gained prominence [40,49].

In line with waste collection, our research group has also explored the realm of edge computing to address waste collection compliance. We have leveraged edge devices such as NVIDIA Jetson modules to enable automatic waste identification on collection trucks using video analytics [50]. The integration of standalone sensors, as discussed in our previous discussion, could complement visual data and contribute to an enhanced real-time compliance model.

Furthermore, in waste collection optimisation, Saha et al. [40] discussed the utilisation of ultrasonic level sensors in dustbins to optimise collection resources. Leveraging the versatility of our control board, we envision that off-the-shelf levelling sensors could seamlessly integrate with our system, making it suitable for such applications.

The scope of our system extends to diverse waste management contexts, including anaerobic digestion (AD). Radu et al. [51] have explored the potential of IoT monitoring solutions for tracking biogas generation in AD reactors. Given the adaptable nature of our platform, it holds promise for similar applications in AD environments.

It is also worth noting that our system is designed for versatility and robustness. The utilisation of a highly robust casing (Pelican, 1300) with IP67 and Def Stan 81-41 accreditation ensures its capacity to operate effectively in extreme or hazardous environments.

In summary, our research has demonstrated great potential for the adaptability of our platform to accommodate a range of sensors and applications. The flexible architecture, combined with its robust casing, positions our system to be applicable in various waste management scenarios across diverse environments.

## 5. Future Work and Summary

### 5.1. Future Developments and Improvements

The device developed during this study was at the prototype stage with its main focus as a proof of principle application system. The practical experience gained throughout this study has allowed scope for future developments/improvements in its design. For instance, a lower cost system could be realised by adapting modern System-on-Chip (SoC) technology with integrated wireless communications [52]. The recent proliferation of the mobile phone industry has given rise to integrated low-cost control and communication devices, with technology summarily available via platforms such as Wixel, Arduino, or Raspberry Pi.

### 5.2. Intelligence Integration and Autonomous Management

For larger-scale deployments, a gateway node [53,54] can be deployed on site with a permanent connection to the Internet via 4/5G. The significance of this can allow for elimination of the GSM module and allow for sites to operate independently from relatively placed remote base stations, in addition to providing longevity of the system without the power demands of GSM activity. An interesting alternative to self-deployed wireless architectures is the availability of wireless LoRa network and data access via The Things Network [55].

Other envisioned developments include the integration of intelligence into the nodes themselves such as delta reporting [56] (i.e., report when a certain threshold is breached) to conserve resources and therefore increase operational lifetime. Additional advances can involve the introduction of the sensory targets. Examples include the development of cost-effective O${}_{2}$[57] and/or CO${}_{2}$ [58] sensors based on LED detection technology [59,60,61]. In addition to cost-effectiveness, LED photometry via the PEDD technique offers improvements in sensitivities and resolution [62].

At this stage of development, we have designed our system as a platform capable of integrating other sensory targets where required [35], which can include water monitoring for leachate targets [63,64,65]. To effectively manage complex systems such as landfill sites, it is envisioned that a more holistic approach will be required with monitoring of several sensory targets and possibly analysed in real time via AI. Overall, the needs/demands of the future application stages will dictate the direction and therefore the optimisation of the sensing approach.

It is well recognised that post-closure landfill management requires sustained monitoring and timely intervention to ensure long-term efficiency and safety [66,67]. Our proposed system significantly contributes to enhancing landfill management by facilitating increased monitoring capabilities and issuing timely alerts to mitigate critical events. Leveraging the GSM module integrated into the system, we can notify stakeholders directly through SMS, calls, or cloud-based platforms, enabling swift corrective actions.

Furthermore, our platform is designed for scalability and adaptability. By building upon the open-source MSPGCC framework, we foresee ample room for further enhancing the intelligence and capabilities of the system to address various scenarios. As the field of artificial intelligence advances, the potential to automate certain aspects of landfill management becomes increasingly promising [68,69]. Our platform can serve as a foundation for achieving this goal. While the full automation and suggestion of corrective actions based on AI may require substantial training data and evolving algorithms, our system offers a platform to collect high-resolution, sensor-rich datasets, enabling the development and validation of more intelligent landfill management strategies.

In the long term, the integration of AI and automation could lead to a more proactive approach in landfill management, optimising resource utilisation and response times. This work opens avenues for harnessing data-driven intelligence to revolutionise landfill management practices and ensure the sustainable operation of landfill sites.

### 5.3. Limitations and Future Directions

Our research contributes to the existing literature by introducing a novel approach to landfill gas monitoring and management. By combining advanced sensing technologies, wireless communication capabilities, and an autonomous data transmission system, we provide a comprehensive solution for improved gas pressure monitoring and early event detection. The developed device fills a gap in environmental monitoring and offers a cost-effective and scalable solution for sustainable waste management [70]. Through extensive field experiments and validation against high-end analytical systems, we demonstrate the reliability, accuracy, and high sampling rate of our device [71]. The implications of our research findings are significant for both landfill operators and environmental practitioners. The real-time monitoring capability of our device enables the prompt detection of critical events such as flare shutdowns, blockages, and pressure increases [38]. This early detection can facilitate timely interventions and preventive measures, minimising the risk of hazardous conditions and improving overall safety in landfill operations. Additionally, the remote access and data visualisation features of our system provide landfill operators with valuable insights into gas dynamics, enabling them to make informed decisions for optimised gas extraction and management strategies [39].

In terms of limitations, we have identified several areas that require further attention. Firstly, regarding energy usage, we acknowledge the need to optimise the device’s power consumption. This can be achieved through the implementation of low-power and sleep modes with precise timing. Additionally, exploring alternative communication networks such as LoRa can reduce the power requirements associated with GSM communications. Incorporating solar panel technology could also extend the device’s operational lifespan. In terms of communications, we recognise the cost implications of using GSM services. As suggested, investigating the feasibility of utilising a less expensive system like LoRa or WiFi is a valid direction for future research. However, we acknowledge the security concerns associated with WiFi and will take those into account in our considerations. Additionally, the limitations imposed by the data packet size in an SMS should be considered when scaling up the sensor network. Lastly, we understand the importance of cost optimisation. As the current system is a prototype, there is room for improvement in terms of the Bill of Materials (BOMs). Conducting a design for manufacture and assembly analysis will help us identify areas for cost reduction and optimise the system’s overall affordability.

Overall, this research introduces a novel, autonomous, and stand-alone device capable of real-time gas pressure monitoring on active landfill sites, offering a comprehensive solution for improved landfill gas management. The device combines advanced sensing technologies, wireless communication capabilities, and an autonomous data transmission system to enable remote access to gas dynamics information via the Internet with the capability of issuing alerts. Through extensive field experiments and validation against high-end analytical systems, this study demonstrates the device’s high sampling rate and its ability to detect critical events such as flare shutdowns or localised blockages. The developed technology presents a cost-effective and scalable approach that fills a gap in environmental monitoring, contributing to more efficient waste management practices and facilitating the early detection and prevention of hazardous conditions in landfills.

## 6. Conclusions

In this paper, we have achieved a significant milestone in the development of a self-contained apparatus for measuring gas pressure levels on active landfill sites and wirelessly transmitting this valuable information for remote access via the Internet. The utilisation of a high sampling rate in our design has provided a wealth of information regarding landfill gas generation, allowing for the detection of critical events such as flare shut down/failure. While event detection is beyond the scope of our study, the integration of supplementary data sources with pressure sensing holds the potential to create an automatic detection system that offers early warnings to prevent catastrophic outcomes.

Furthermore, we have conducted a comprehensive validation of our findings on a global scale by comparing them to the data obtained from a high-end analyser system located at the main flare. This validation process has also revealed the ability of our system to detect instances when the main flare was offline, thereby enhancing the quality assurance protocols of landfill sites. Additionally, our study has demonstrated that localised pressure changes can serve as indicators of potential blockages and/or pressure increases, which, under certain conditions, may signify the early stages of underground fires or methane explosions. The deployment of these relatively low-cost sensing devices has the potential to prevent disastrous consequences by providing localised information along the extraction network.

Compared to conventional SCADA systems, our platform offers several distinct advantages, including higher sampling rates, lower cost, the ability to operate without mains power, and better compatibility with economies of scale. Overall, this study has provided a comprehensive understanding of the capabilities of our stand-alone device and its potential for improving landfill management systems.

In conclusion, our research significantly contributes to the field of landfill gas monitoring and management by introducing an advanced IoT pressure monitoring system. Through a thorough comparison with existing work and a detailed discussion of our contributions to theory, knowledge, and literature, as well as the implications for practice, we aim to provide a comprehensive understanding of the novelty and significance of our research [34,38,39,40,70,71].

## Figures and Tables

**Table 1:** Comparison of detected events from both our sensing system and the SCADA system for Location #2.

Event Description	Sensing System	SCADA
Total Number of Events	14	10
Sampling Period	4 min	10 min
Maximum Duration	2 h 31 min	2 h 29 min
Minimum Duration	4 min	10 min
Average Duration	49 min	1 h 6 min

**Table 2:** Global statistical representation of the recorded pressure readings.

Description	Value
Total Number of Measurements	20,043
Sampling Period	4 min
Number of Measurements Per Day	360
Maximum Recorded Measurement	0.96 mBar
Minimum Recorded Measurement	−52.84 mBar
Average of the Recorded Measurements	−25.62 mBar
Percentage of Measurements Above −20 mBar	13.65%

**Table 3:** Statistical representation of the detected events by the prototype sensing system for the entire deployment duration.

Event Description	System
Total Number of Events	21
Maximum Duration	21 h
Minimum Duration	4 min
Average Duration	1 h 50 min

**Table 4:** Direct comparison of devices reported in the literature.

Criteria	Mabrouki et al. [32]	Venkatesan et al. [33]	Fay et al. [35]	This Study
Sensing Environment	Landfill Site	Laboratory	Landfill Site	Landfill Site
Sensory Targets	CO${}_{2}$, CH${}_{4}$, H${}_{2}$S, O${}_{2}$, N${}_{2}$	Temperature, Soil Moisture, CO${}_{2}$, CH${}_{4}$	Temperature, Humidity, CO${}_{2}$, CH${}_{4}$	Pressure
Monitored Pressure	No	No	No	Yes
Calibration/Validation	No	No	Yes	Yes
Data Compared to In-field Instrumentation	No	No	No	Yes
Wireless Communications	WiFi	WiFi	GSM	GSM
Electronics Platform	Arduino	Arduino	Ad Hoc	Ad Hoc
Sampling Period	Unknown	Unknown	6 h	4 min

## Data Availability

Restrictions apply to the availability of these data. Data was obtained from a private landfill site and can be requested from the EPA in the first instance.

## Abbreviations

The following abbreviations are used in this manuscript:

IoT	Internet of Things
CO${}_{2}$	Carbon Dioxide
CH${}_{4}$	Methane
H${}_{2}$S	Hydrogen sulfide
O${}_{2}$	Oxygen
N${}_{2}$	Nitrogen
EPA	The Environmental Protection Agency
SCADA	Supervisory Control and Data Acquisition System
